# Identification and Validation of a Prognostic Immune-Related Alternative Splicing Events Signature for Glioma

**DOI:** 10.3389/fonc.2021.650153

**Published:** 2021-05-13

**Authors:** Minjie Wang, Zijie Zhou, Jianglin Zheng, Wenxuan Xiao, Jiameng Zhu, Chaocai Zhang, Xiaobing Jiang

**Affiliations:** ^1^ Department of Neurosurgery, Union Hospital, Tongji Medical College, Huazhong University of Science and Technology, Wuhan, China; ^2^ Department of Neurosurgery, Hainan General Hospital/Hainan Affiliated Hospital of Hainan Medical University, Haikou, China

**Keywords:** glioma, alternative splicing, immune microenvironment, prognosis, signature

## Abstract

**Background:**

Glioma is the most common malignant brain tumor in adults, with its tumor-promoting immune microenvironment always being intricate to handle with. Amounts of evidence has accumulated to suggest that alternative splicing (AS) is related to tumor immune microenvironment. However, comprehensive analysis of immune-related AS events and their clinical significance are still lacking in glioma.

**Methods:**

AS events and transcriptome data of 653 glioma patients were downloaded online. ssGSEA was performed on transcriptome data of 653 patients to divided them into low, medium and high immune cell infiltration groups. Immune-related AS events were filtrated based on this grouping. Then lasso Cox regression analysis and multivariate Cox regression analysis were done to achieve an immune-related AS events prognostic signature for glioma. Kaplan-Meier analysis, ROC analyses, univariate Cox regression and multivariate Cox regression were performed to reveal the independent prognostic role of this signature. Meanwhile, a nomogram was constructed to achieved better prognostic value for glioma patients. Besides, functional enrichment analyses and correlation analyses with immune cells infiltration were used to validated the immune-related characteristic of this signature.

**Results:**

36 immune-related AS events were achieved based on the grouping mentioned above. A nine-immune-related alternative splicing event signature was built for glioma patients. This signature showed an independent prognostic value and a nomogram containing gender, age, Karnofsky performance score, grade, IDH status, MGMT promoter status and risk score derived from the signature was constructed with a higher predictive ability for overall survival. Association with the infiltration of immune cell subtypes was validated and functional enrichment analysis found that the signature was mainly enriched in immune-related and pro-tumor functions.

**Conclusion:**

Our research presented all immune-related AS events in glioma, identified an immune-related prognostic AS events risk model and a nomogram was constructed to predict the prognosis individually and more precisely. Tight connection was verified between this signature and clinical characteristics. Also, immune cells infiltration and immune checkpoints expression level were proved to link to risk scores, which enhanced the understanding of relationship between AS events and glioma immune microenvironment, firstly revealing the potential role of AS in immunotherapy of glioma.

## Introduction

Glioma is the most common primary brain tumor which accounts for 50% to 60% in the central nervous system (1). Glioblastoma (GBM), as the most malignant type of glioma, has the worst outcome with the median survival time only approximately 15 months (2, 3). Recently, immune suppressive microenvironment, a complex system consisting of tumor cells and non-tumor immune cells, was proved as a key factor for tumor development (4). In glioma, several kinds of infiltrated immune cells have been proved to enhance the aggressiveness of cancer such as tumor associated macrophages (TAMs), regulatory T cells (Tregs) and myeloid-derived suppressor cells (MDSCs) (5, 6). Hence, an improved understanding of the molecular mechanism underlying the immune characteristics of glioma is urgently needed.

Alternative splicing (AS), a ubiquitous process by which a single pre-mRNA can generate diverse mature mRNAs and then expand the protein diversity, providing the potential for functional and regulatory complexity in cells (7). Genome-wide studies showed that 90% to 95% of human genes undergo some level of alternative splicing, and almost one-third of them were proved to generate multiple protein isoforms (8, 9). Studies demonstrated the physiological contribution of AS to the tissue-identity acquisition, organ development and tissue physiology; meanwhile, studies of AS also demonstrated its involvement in multiple pathologies, including cancer (10). Human cancers can take advantage of aberrant AS to develop, grow and progress into therapy-resistant tumors (11). Recently, the importance of AS on tumor immunity is gradually being widely supported and increasing analysis of AS events have demonstrated the independent oncogenic effects that could be relevant to the suppressive immune microenvironment in cancers (12–14).

To date, several prognostic signatures derived from AS events has been identified in glioma (15, 16). However, to our knowledge, researches on immune-related AS events in glioma and their prognostic value are still lacking. Therefore, we aimed to establish a prognostic signature with immune-related AS events which can exactly predict the prognosis of glioma. In this study, we downloaded transcriptome and clinical data of 653 glioma patients from the Cancer Genome Atlas (TCGA) and obtained the corresponding data of AS events from the SpliceSeq database. Then, we conducted a comprehensive profiling of immune-related AS events in glioma and identified a prognostic immune-related AS events signature, laying foundation for immunotherapy and prognosis prediction.

## Materials and Methods

### Data Collection and Pretreatment

The RNA-seq information and the clinical and pathological variables of 653 GBM (glioblastoma) and LGG (low grade glioma) samples were obtained from the TCGA database. The data of AS events corresponded with the samples were obtained from TCGA SpliceSeq database (https://bioinformatics.mdanderson.org/TCGASpliceSeq/PSIdownload.jsp). AS events were automatically detected and quantified using the percent-spliced-in (PSI) metric based on long (L) and short (S) forms of all splicing events presents (PSI = L/(L+S)). Briefly, for each splicing event in one given gene, a PSI value is the ratio of normalized read counts indicating inclusion of a transcript element over the total normalized reads for that event (both inclusion and exclusion reads) with the quantization interval (0-1).

### Filtration of Immune-Related AS Events

Firstly, aiming to stratify the 653 patients into subgroups according to the level of immune infiltration, we used the packages “GSVA” and “hclust” of R software based on ssGSEA method. Subsequently, the samples were separately into three immune subtype groups: high-immunity group (n = 107), medium-immunity group (n = 134), and low-immunity group (n = 412). To validate that the immune subgroups were feasible to reveal the immune characteristics of the 653 samples, we calculated the stromal score, immune score, estimate score and tumor purity using the ESTIMATE algorithm. ESTIMATE is a method that uses gene expression signatures to infer the fraction of stromal and immune cells in tumor samples. It outputs stromal, immune, and ESTIMATE scores by performing ssGSEA based on the signatures related to stromal tissue and immune cell infiltration. The formula for calculating ESTIMATE-based tumor purity was developed as follows: tumor purity = cos (0.6049872018 + 0.0001467884 * ESTIMATE score) and the results of ESTIMATE were exhibited in the form of a heatmap and boxplots (17). Meanwhile, the expression level of human leukocyte antigen (HLA) and CD274 (PD-L1) of the three subtype groups were displayed by the virtue of R software. Finally, the differences of the immune cells compositions in three immune subgroups were revealed with a boxplot. Based on the immune-related subgroups, by using “edgeR” package, AS events whose PSI value were significantly different between three immune subgroups were respectively filtrated out with the criteria (|log2FC|>1 and p <.05). A venn plot was then constructed to show the intersectional part in both subgroups. Taken together, the AS events filtrated were considered as immune-related AS events.

### Establishment and Evaluation of the Signature

Univariate Cox regression analysis was then implemented to identify OS (overall survival)-related AS events with *p* <.05 as the criterion. Using lasso Cox regression with 10-fold-cross validation and multivariate Cox regression, a signature involving immune-related AS events and correspondent coefficients were finally completed.

The formula to calculate the risk score for the diffuse glioma patients each was:

Risk score=PSI1∗β1+PSI2∗β2+…+PSIi∗βi

where PSIi was the PSI value of the immune-related AS event, βi was the correspondent regression coefficient. Upon the formula was obtained, the correspondent risk score was figured out and the samples were classified into high- and low-risk subgroups with the median risk score serving as cut-off value. Subsequently, Kaplan-Meier survival analysis was conducted to compare the OS between the risk subgroups. The ROC analysis was conducted to evaluate the ability to predict prognosis of diffuse glioma. The correlation between risk score and clinical variables such as age, gender, Karnofsky performance score, IDH status and MGMT promoter status, were analyzed by the virtue of the correlation analyses. The packages of R software used in this section were listed here: “survival,” “UpSetR,” “glmnet,” “survminer,” “survivalROC,” “limma,” and “ggpubr.”

### Construction and Evaluation of Nomogram

Univariable and multivariable Cox regression analysis was applied to access the relationship between risk score and age, gender, grade, and clinical stage. A nomogram was established by using TCGA dataset. The calibration plots showed the prognostic predictive accuracy of the nomogram and the C-index was also calculated. C-index is the probability that the predicted outcome be consistent with the actual observed outcome, evaluating the predictive efficiency of the model. It has a value between 0.5 and 1, demonstrating progressive predictive efficiency of model respectively: 0.50 to 0.70 (low), 0.71 to 0.90 (intermediate), and >0.90 (high). R package “rms” was applied during these analyses. The predicting-performance of the nomogram and other predictive factors (risk score, age, WHO grade, IDH mutant state, Karnofsky performance score and MGMT promoter status) for 1/3/5-year OS were accessed using ROC curves.

### Functional Enrichment Analysis

To analyze the function enrichment of the differentially expressed genes (DEGs) between risk subgroups, the Gene Ontology (GO) enrichment analysis and Kyoto Encyclopedia of Genes and Genomes (KEGG) analysis were performed. The DEGs between risk subgroups were filtrated with the enrollment criterion (|log2FC | > 1 and *p* < 0.05).

### Correlation Analysis

Spearman correlation analyses were implemented to analyze the correlation between risk score and CD8+ T-cell, T-cell regulatory (Tregs), NK cells activated, macrophage (M1, M2), and neutrophil immune infiltration data using CIBERSORT deconvolution algorithm with R software. And Spearman correlation analyses between risk score and expression of immune checkpoints were also performed with the same manner after tests for normal distributions (Kolmogorov-Smirnova test and Shapiro-Wilk test).

### Statistical Analysis

All statistical analyses and figures were accomplished and drawn respectively by virtue of R software (version 4.0.3). The packages used in this section included “limma,” “ggplot2,” “ggpubr,” “ggExtra,” and “survival.” Significant differences were identified if met the standard of *p*-value <.05 (two-side). “***” means *p*-value < 0.001, “**” means *p*-value < 0.01, “*” means *p*-value < 0.05.

## Results

### Grouping of Diffuse Glioma Samples Based on the Immune Subtypes

**Figure 1:**
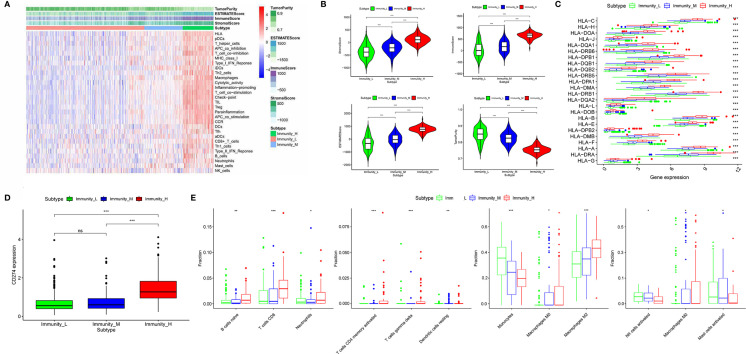
Classification of diffuse glioma samples based on the immune subtypes. **(A)** 653 samples divided into three groups according to the results of the immune infiltration. **(B)** Validation of the grouping with ESTIMATE algorithm. **(C)** The expression of HLA family between three immune cell infiltration groups. **(D)** The expression of CD274 (PD-L1) between three immune cell infiltration groups. **(E)** Comparison of several kinds of immune cells between three immune cell infiltration groups. (“*” means p-value < 0.05, “**” means p-value < 0.01, “***” means p-value < 0.001, “ns” means not significantly different).

653 diffuse glioma samples were obtained from the TCGA database. The infiltration of immune cells was assessed using the ssGSEA method with transcriptome data of the 653 samples. The richness of multiple immune cell types of glioma was evaluated under the participation of twenty-four immune-related terms. To strengthen the accuracy, we divided the 653 samples into three groups: the high immune cell infiltration group (n = 107), the medium immune cell infiltration group (n = 134) and the low immune cell infiltration group (n = 412) according to the results of the immune infiltration using unsupervised hierarchical clustering algorithm (**Figure 1A**). To make sure that the grouping method was feasible, we applied the ESTIMATE algorithm to evaluate immune score, stromal score, ESTIMATE score, and tumor purity based on the expression profile of glioma (**Figure 1B**). Comparing the immune score, stromal score, ESTIMATE score, and tumor purity of the three subtypes, we found that there was a significantly positive correlation between immune cell infiltration groups and ESTIMATE score, and the same was true of immune score and stromal score (*p* <.01). As a matter of course, there was a significantly negative correlation between immune cell infiltration groups and tumor purity (*p* <.01). Meanwhile, the expression of HLA family and CD274 (PD-L1) in the low & medium infiltration groups was lower than that in the high immune cell infiltration group, and the difference was significant (*p* <.01) (**Figures 1C, D**). The next section was concerned with the CIBERSORT method, it was established that the kind of immune cells in the high immune cell infiltration group was more plentiful than that in low or medium ones (**Figure 1E**). Consequently, we demonstrated that our standard of classification was feasible to continue our following analysis.

### Identification of the Immune-Related AS Events From Diffuse Glioma Samples

**Figure 2:**
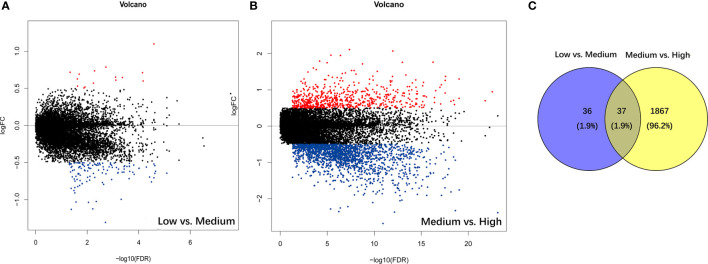
Identification of immune-related alternative splicing events. **(A)** Differential AS events between low and medium immune cell infiltration groups. **(B)** Differential AS events between medium and high immune cell infiltration groups. **(C)** Venn plot for the ultimate immune-related AS events.

The PSI difference of AS events was analyzed under the standard of |log2FC| > 0.5 and *p* < 0.05. Comparing to the low immune cell infiltration group, there were 73 significantly different AS events, containing 11 up-regulated and 62 down-regulated ones in medium immune cell infiltration group (**Figure 2A**). Respectively, compared with medium immune cell infiltration group, there were 1904 significantly different AS events, with 472 up-regulated and 1432 down-regulated ones in high immune cell infiltration group (**Figure 2B**). As shown in the Venn plot, there were 37 significant different AS events which were sorted out by a two-way Venn analysis (**Figure 2C**). Consequently, 36 out of 37 immune-related AS events were standard-compliant as one AS event was removed as the variation trend was not consistent during the enrollment process, and the other 36 immune-related AS events were finally selected for our following research.

### Identification and Assessment of Nine-Immune-Related AS Events in Glioma

**Figure 3:**
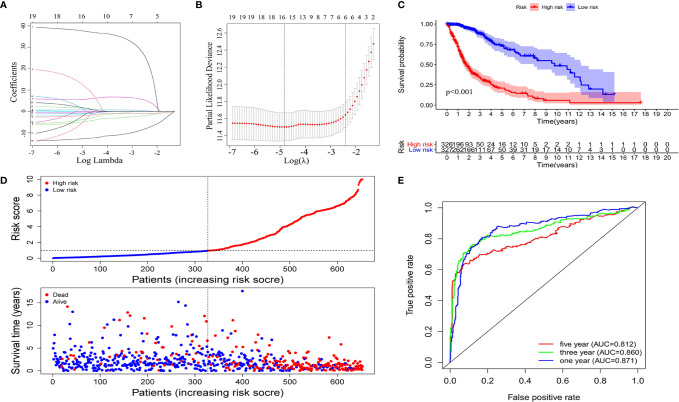
Construction of the prognostic signature based on the immune-related AS events. The LASSO regression analysis identified nine-immune-related prognostic AS events **(A, B)**. **(C)** Kaplan-Meier curves of low and high-risk groups. **(D)** Risk curves and scatter plots for each glioma sample. **(E)** Calculation the AUC of 1-year, 3-year and 5-year survival rate according to the ROC curve.

Using univariate Cox regression analysis, the results revealed that the 36 selected AS events were all OS (overall survival)-related (*p* < 0.05) (**Table S1**). To decrease the phenomenon of over-fitting of prognostic signature, lasso Cox regression analysis was applied to these AS events and 15 AS events were further selected. Meanwhile, the optimal value of the penalty parameters was identified through 10 rounds of cross-validation (**Figures 3A, B**). Using stepwise multiple Cox regression analysis, nine AS events were further filtrated from the above 15 AS events, including IFITM3|13647| AP, PIK3R2|48396|AT, IL1RAP|68106|AT, MYO10|71604|AT, FCER1G|8600|AT, ARHGAP15|55490|AT, CARD6|71874|AT, SCPEP1|42600|AA, and ATP1B3|67084|ES (**Table S2**). Risk scores were calculated separately for each sample based on the PSI data of these nine AS events. The formula of risk score = −14.36*IFITM3|13647|AP−2.80*PIK3R2|48396|AT−6.70*IL1RAP|68106|AT+43.35*MYO10|71604|AT+20.32*FCER1G|8600|AT−5.25*ARHGAP15|55490|AT−2.39*CARD6|71874|AT+2.11*SCPEP1|42600|AA+2.95*ATP1B3|67084|ES. The 653 samples were then divided into high- and low-risk groups based on the data of calculated risk scores. Kaplan-Meier curves showed that the overall survival (OS) of samples in the high-risk group was worse than those in the low-risk group, indicating that the risk score was a valid prognostic index (*p* < 0.001) (**Figure 3C**). To assess the risk scores and survival status of each glioma sample, we plotted the generated risk curves and scatter plots separately, and found that samples from the high-risk group had higher risk coefficients and mortality rates than those from the low-risk group (**Figure 3D**). The time-dependent receiver operating characteristics (ROC) analysis revealed that the risk score was an OS-predicting index as the AUC value were 0.871, 0.860, 0.812 separately for 1, 3, and 5 years (**Figure 3E**).

### Stratification Analysis of the Prognostic Signature Based on Clinical Features

**Figure 4:**
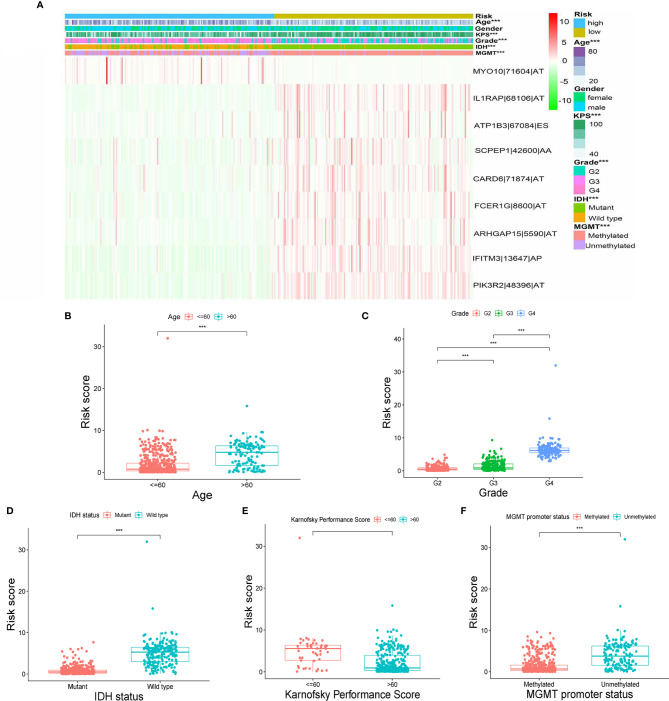
Correlation between signature and clinical features. **(A)** Heatmap for nine AS events with clinical features. **(B–F)** Correlations between risk scores and age, grade, IDH status, Karnofsky performance score and MGMT promoter status. (“***” means p-value < 0.001).

The heatmap of the PSI data of these nine AS events in glioma samples show that eight AS events are down-regulated in the high-risk subtype while the MYO10|71604|AT is up-regulated in high-risk subtype (**Figure 4A**). Moreover, as the heatmap and boxplots shown (**Figures 4A–F**), the high/low subtypes were significantly correlated with age, Karnofsky performance score, grade, IDH status and MGMT Promoter Status with the p value were less than 0.001. To illustrate more details, the patients of high-risk subtype had lower Karnofsky performance score, higher age, higher WHO grade, meanwhile, tended to have mutant IDH and unmethylated MGMT Promoter (**Figures 4B–F**). Further, we validated the prognosis-predicting ability of risk signature in subgroups by age, grade, IDH status and MGMT promoter status (without Karnofsky performance score due to censored data) with Kaplan-Meier curves. The results all demonstrated worse prognosis in the high-risk groups (**Figure S1**).

### Construction and Validation of a Nomogram

**Figure 5:**
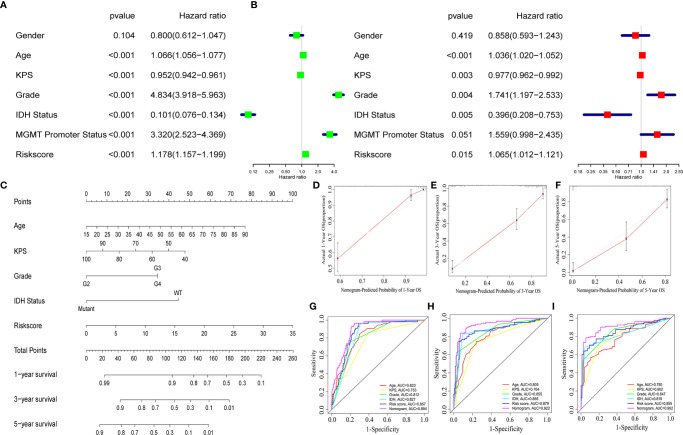
A nomogram based on the AS signature and clinical features. Univariate **(A)** and multivariate **(B)** Cox regression analyses and the model revealed satisfactory prognostic efficiency independent of clinical factors. **(C)** A nomogram based on the data of risk score, age, Karnofsky performance score, grade and IDH status. **(D–F)** Calibration plots revealed the concordance in predicting the OS of 1, 3, and 5 years. **(G–I)** ROC curves were used to evaluate the predictive ability of nomogram and other predictors.

To identify whether the selected nine-immune-related AS events can be qualified for prognostic signature of glioma, we conducted univariate and multivariate Cox regression analyses and the model revealed satisfactory prognostic efficiency independent of clinical factors, for instances, gender, age, Karnofsky performance score, grade, IDH status, and MGMT Promoter Status, as the hazard ratio (HR) of risk score and the 95% confidence interval (CL) were 1.178 and 1.157 to 1.199 in univariate Cox regression (*p* < 0.001) (**Figure 5A**), and the results in multivariate Cox regression analysis were 1.065 and 1.012 to 1.121 (*p* < 0.001) (**Figure 5B**). To make our prognostic signature more applicable for clinical use, we constructed a nomogram based on the data of risk score, age, Karnofsky performance score, grade, and IDH status to predict the OS of 1, 3, and 5 years (**Figure 5C**). Meanwhile, the nomograms showed excellent concordance in predicting the OS of 1, 3, and 5 years as the Calibration plots revealed (**Figures 5D–F**). Using time-dependent receiver operating characteristics (ROC) analysis, we found that the nomogram based on nine-immune-related AS events showed excellent sensitivity and specificity for the prognosis-prediction of glioma, as the area under the curve (AUC) of the nomogram were 0.894, 0.922, 0.902 for 1, 3, and 5 years (**Figures 5G–I**). Meanwhile, we calculated the C-index of the nomogram and the value was 0.852 (reflecting upper intermediate predictive efficiency). On this basis, we validated the prognostic predictive ability of the nomogram in subgroups by age, grade, IDH status, and MGMT promoter status (without Karnofsky performance score due to censored data) with AUCs. The results showed strong prognosis-predicting ability in all subgroups (**Figure S2**). These results together revealed the satisfactory prognostic efficiency of the nine immune-related AS events for glioma, which was a bright spot for the prognosis for glioma patients.

### Enrichment Analysis and Correlation With Immune Infiltration Cells Subtype

**Figure 6:**
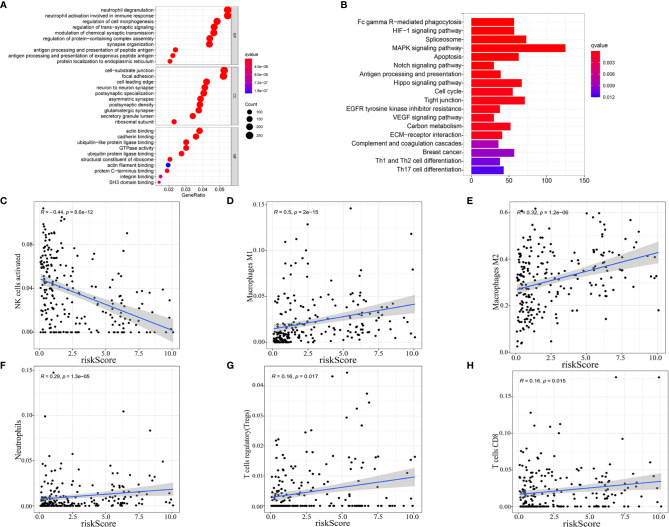
Enrichment analysis and correlation with immune infiltration cells subtype. Gene ontology (GO) enrichment **(A)** and Kyoto Encyclopedia of Genes and Genomes (KEGG) **(B)** analysis based on the differentially expressed genes (DEGs) between high-risk score and low-risk score groups. **(C–H)** Correlation between immune cell infiltration and risk score.

As our nine selected AS events were immune-related, gene ontology (GO) enrichment analysis were then performed based on the differentially expressed genes filtrated from the risk score subtypes and indicated that the differentially expressed genes were mainly enriched in projects linked to immunity, for instances, neutrophil degranulation, neutrophil activation involved in immune response, antigen processing and presentation of peptide antigen, antigen processing, and presentation of exogenous peptide antigen (**Figure 6A**). Consequently, the bar plot of Kyoto Encyclopedia of Genes and Genomes (KEGG) analysis revealed the enrichment in Fc gamma R−mediated phagocytosis, HIF-1 signaling pathway, MAPK signaling pathway, and so on (**Figure 6B**), which demonstrated the close connection between the differentially expressed genes and immunity together with tumor aggressiveness. Consequently, correlation analysis was performed to identify the correlation of our immune-related prognostic signature and the subtypes of immune cell infiltration in glioma. The spearman correlation coefficients of NK cells activated cells, macrophages M1 cells, macrophages M2 cells, neutrophils cells, T cells regulatory (Tregs) cells, and T cells CD8 cells with risk score were, respectively −0.44, 0.5, 0.32, 0.29, 0.16, and 0.16 (normal distribution tests’ results in **Table S3**) (**Figures 6C–H**). In view of the positive relation between risk score and CD8+ T cell infiltration, the distribution of immune checkpoints was stepwise researched and the results showed that high-risk score signify high expression of immune checkpoints (normal distribution test results in **Table S4**) (**Table S5**). Moreover, according to the results in stratification analysis that the risk scores were strongly related to IDH status, we individually evaluated the correlation of risk scores with immune infiltration cells subtype in IDH-wild type and IDH-mutant gliomas to maximally reduce the bias derived from IDH status. As the results showed in **Figure S3**, risk scores were positively related to infiltrations of macrophages M2 cells and neutrophils and negatively related to infiltrations of NK cells activated and T cells CD4 memory resting in IDH-wild type gliomas (*p* < 0.05); while risk scores were positively related to infiltrations of macrophages M1 cells, NK cells resting and negatively related to infiltrations of NK cells activated and mast cells activated in IDH-mutant gliomas (*p* < 0.05). Meanwhile, we performed the similar analyses in different molecular classifications of IDH-mutant gliomas: oligodendroglioma, oligoastrocytoma and astrocytoma. In oligodendroglioma, risk scores were positively related to infiltrations of macrophages M1 cells and dendritic cells resting (*p* < 0.05). In oligoastrocytoma, risk scores were positively related to infiltrations of NK cells resting and mast cells resting and negatively related to infiltrations of mast cells activated (*p* < 0.05). In astrocytoma, risk scores were positively related to infiltrations of macrophages M1 and NK cells resting and negatively related to infiltrations of NK cells activated (*p* < 0.05) (**Figure S3**). These results established that AS events prognostic signature for glioma was exactly linked with the immune microenvironment.

## Discussion

Glioma is a refractory tumor in central nervous system, and almost all patients suffer recurrence or drug resistance (18). More efficient methods are urgently needed for glioma therapy. Previous research has established that tumor immune microenvironment (TIME) can largely influence the clinical outcomes of glioma (19–21). As focus had been put on intra-tumor immune cells, different subtypes of intra-tumor immune cells revealed brand-new functions on tumor cells. For instance, Liang et al. verified that neutrophils can accelerate the progression of glioblastoma in a S100-dependent manner and served as a tumor-induced element (22). On the opposite, dendritic cells (DCs) had been found available to act as an autologous tumor vaccine to play a vital role in anti-tumor effects, which enhanced the median survival time of glioblastoma patients to 31.4 months compared to 14.6 months of previous conventional therapy using radiotherapy and adjuvant temozolomide as reported (23–25). Hence, immunotherapy, as a potential treatment in improving TIME, offers the possibility of extending lifespan of glioma patients.

New suitable antigen, mainly derived from mutation, is the key factor in immunotherapy (12, 26). Meanwhile, alternative splicing also contributes to protein complexity and can also generate possible neoepitopes that are often overlooked by researchers (27). In glioma, tumor mutational burden is relatively low and patients’ responses to immunotherapy vary a lot (28). We infer that alternative splicing may regulate the tumor immunity to a great degree and the splicing-derived neoepitope might be used as a prognostic indicator for response to immunotherapy. Thus, we performed a comprehensive exploration for the relationship between alternative splicing and immune-related feature in glioma.

In this study, the entire 36 immune-related prognostic AS events in glioma were strictly screened in TCGA splicing events database for the first time, and the prognostic signature with nine-immune-related AS events in glioma was purposely identified. The results demonstrated the ability of risk signature in predicting the 1-, 3- and 5-year survival of glioma patients with ROC curves. In addition, a nomogram that combined the risk signature and clinicopathological factors (age, Karnofsky performance score, grade and IDH1 status) was established to predict 1-, 3- and 5-year survival rate of glioma patients. Area under ROC curves and calibration plots showed an outstanding performance of the nomogram. Functional analysis and immune cell infiltration analysis once again validated that the risk signature was related to immune response and tumor microenvironment remodeling (29). It is noteworthy that when we turn to our nine-immune-related AS events, some of the corresponding genes turned out to be closely related to the features of glioma. For instances, Wang et al. reported that IFITM3 can promote TGF-beta induced invasion of glioma through IFITM3/STAT3 axis (30); Li et al. reported that IL1RAP participate in the progression of glioma by affecting the synapse development and the differentiation of neuronal cells (31). Meanwhile, several issues that arose in this study must be addressed. First, the study was based solely on online database sources. There is no cross validation for the results, which is certainly a limitation in this study. It is necessary to validate the results using other datasets and experiment in the future. Second, though we discovered that alternative splicing is an important process in glioma immunity, its detailed relationship with AS remains unclear. Thus, this requires further research.

## Conclusion

Our study identified nine-immune-related alternative splicing events that were associated with glioma survival. The nine-immune-related AS events prognostic signature for glioma was associated with the immune-related functions and infiltration of immune cells, which may be used as an indicator for immunotherapy efficiency in the future.

## Data Availability Statement

The data sets presented in this study can be found in online repositories. The names of the repository/repositories and accession number(s) can be found below: https://www.ncbi.nlm.nih.gov/, https://portal.gdc.cancer.gov/.

## Author Contributions

MW provide the idea of this manuscript. ZZ and CZ wrote the manuscript. JMZ and WX drew the figures and tables. XJ and JLZ made some revisions of the review. All authors contributed to the article and approved the submitted version.

## Funding

This work is supported by the National Natural Science Foundation of China (grants 81272778 and 81974390 to XJ).

## Conflict of Interest

The authors declare that the research was conducted in the absence of any commercial or financial relationships that could be construed as a potential conflict of interest.

## Supplementary Material

Supplementary Figure 1 Validation of the prognosis-predicting ability of risk signature in subgroups by age, grade, IDH status and MGMT promoter status with Kaplan-Meier curves. **(A)** Subgroups by age. **(B)** Subgroups by IDH status. **(C)** Subgroups by MGMT promoter status. **(D)** Subgroups by grade.

Supplementary Figure 2 Validation of the prognosis-predicting ability of the nomogram in subgroups by age, grade, IDH status and MGMT promoter status with the area under the curve (AUC). **(A)** Subgroups by age. **(B)** Subgroups by IDH status. **(C)** Subgroups by MGMT promoter status. **(D)** Subgroups by grade.

Supplementary Figure 3 Exploring the relationship between risk scores and immune cell infiltration in different subgroups. **(A)** IDH-wild type subgroup. **(B)** IDH-mutant subgroup. **(C)** IDH-mutant oligodendroglioma subgroup. **(D)** IDH-mutant oligoastrocytoma subgroup. **(E)** IDH-mutant astrocytoma subgroup.
