# Supplementary figures and images for: Identification and Validation of a Prognostic Immune-Related Alternative Splicing Events Signature for Glioma
Source: Front Oncol. 2021 May 13;11:650153. doi: 10.3389/fonc.2021.650153 (PMC8155679; doi:10.3389/fonc.2021.650153)

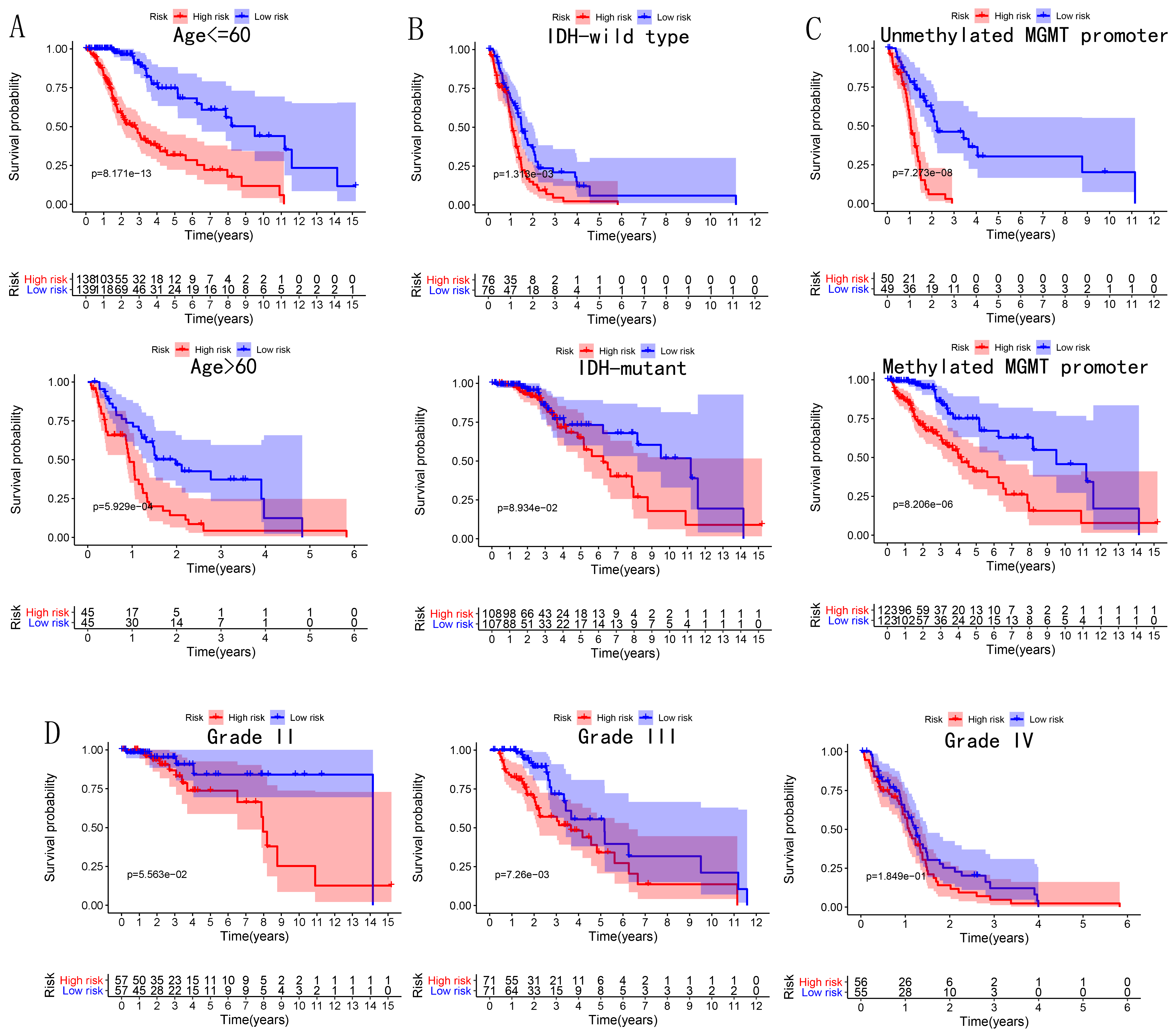

Supplement: Supplementary Figure 1 — Validation of the prognosis-predicting ability of risk signature in subgroups by age, grade, IDH status and MGMT promoter status with Kaplan-Meier curves. (A) Subgroups by age. (B) Subgroups by IDH status. (C) Subgroups by MGMT promoter status. (D) Subgroups by grade. [file Image_1.tif]

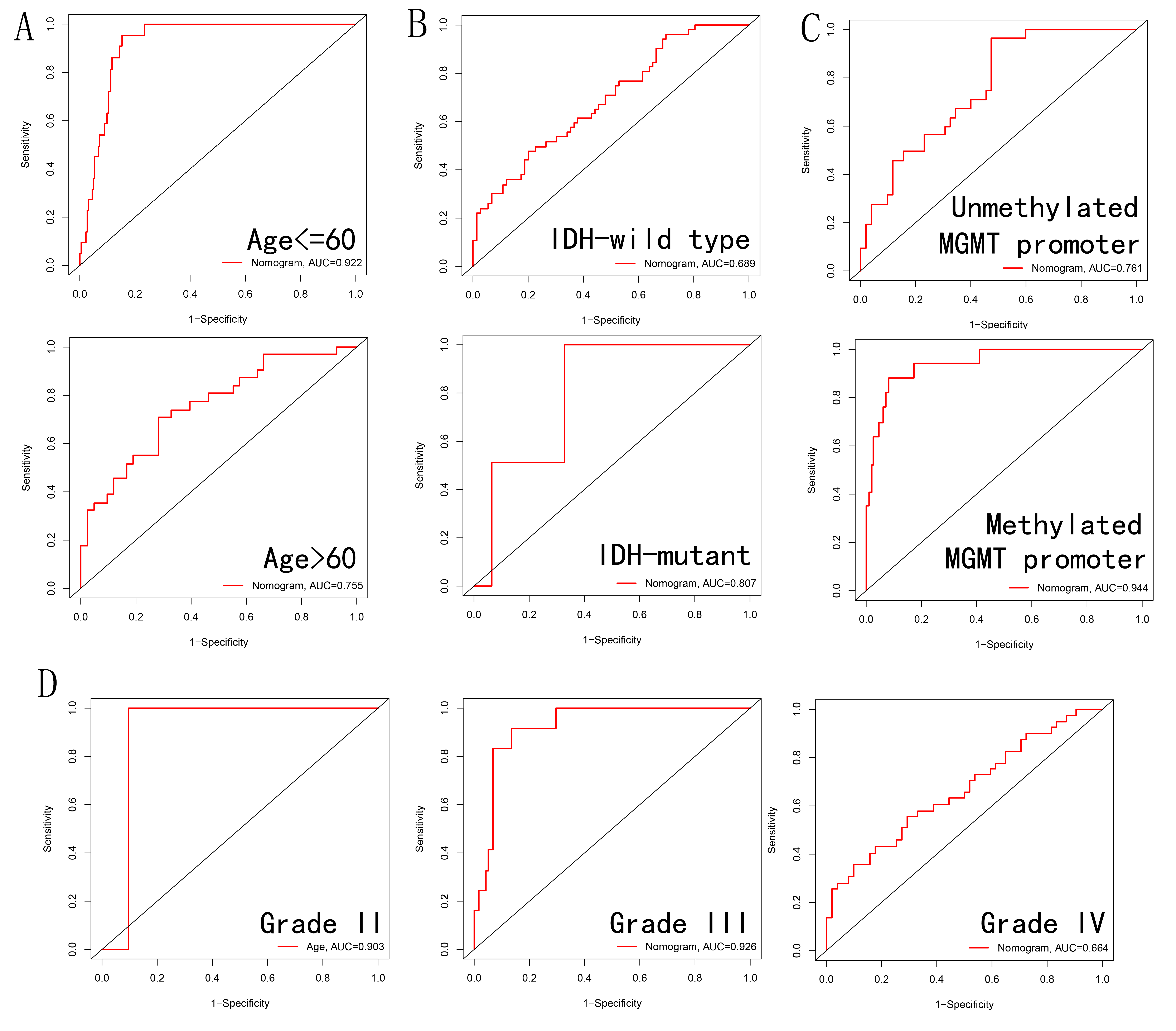

Supplement: Supplementary Figure 2 — Validation of the prognosis-predicting ability of the nomogram in subgroups by age, grade, IDH status and MGMT promoter status with the area under the curve (AUC). (A) Subgroups by age. (B) Subgroups by IDH status. (C) Subgroups by MGMT promoter status. (D) Subgroups by grade. [file Image_2.tif]

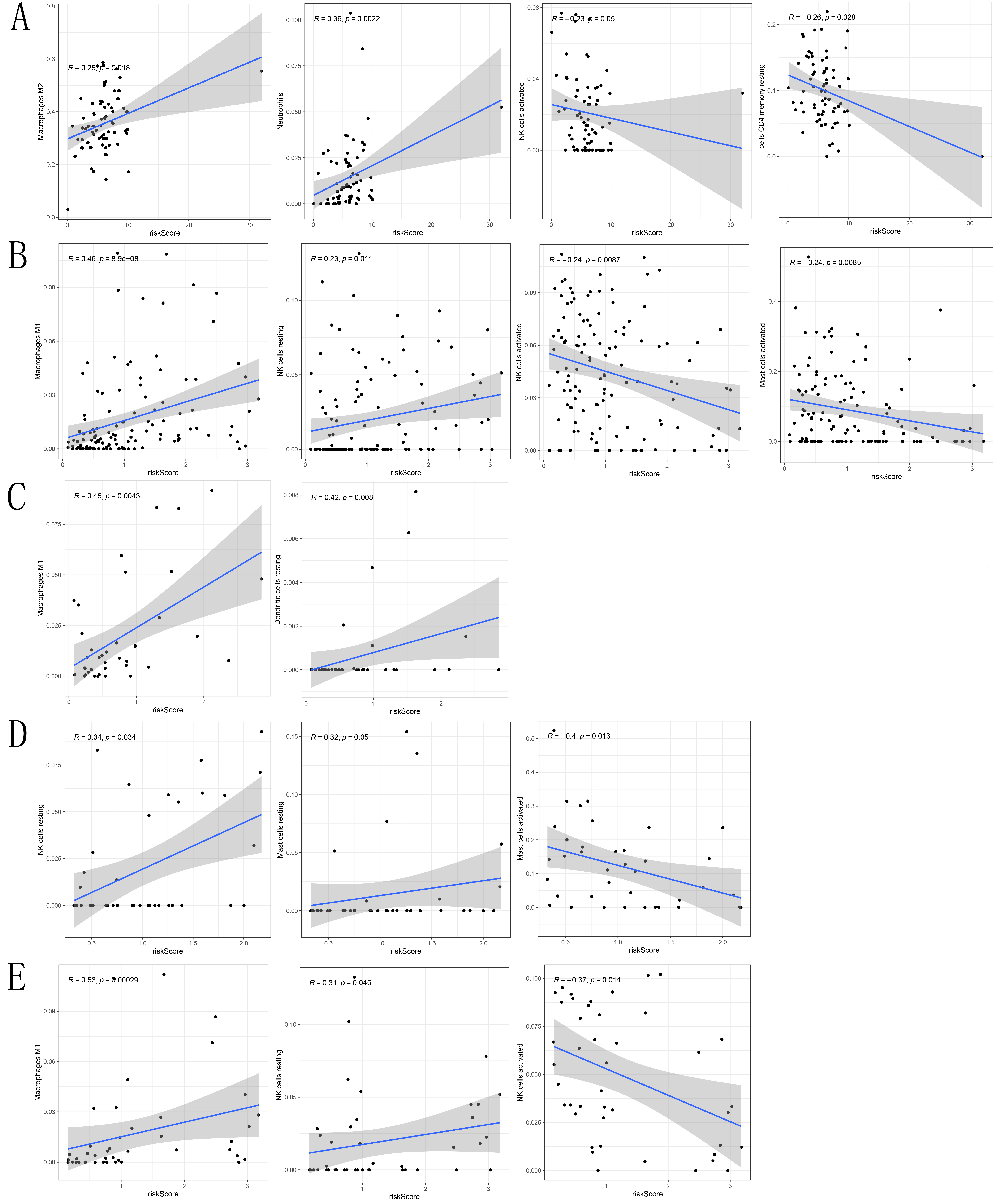

Supplement: Supplementary Figure 3 — Exploring the relationship between risk scores and immune cell infiltration in different subgroups. (A) IDH-wild type subgroup. (B) IDH-mutant subgroup. (C) IDH-mutant oligodendroglioma subgroup. (D) IDH-mutant oligoastrocytoma subgroup. (E) IDH-mutant astrocytoma subgroup. [file Image_3.tif]
