# Supplementary material for: Identification and Validation of a Prognostic Immune-Related Alternative Splicing Events Signature for Glioma
Source: Front Oncol. 2021 May 13;11:650153. doi: 10.3389/fonc.2021.650153 (PMC8155679; doi:10.3389/fonc.2021.650153)
Supplement: Supplementary file 4 [file DataSheet_1.doc]

**Table S1.** Full results of univariate Cox regression analysis.

| **Id** | **Z-score** | **HR** | **HR.95L** | **HR.95H** | **pvalue** |
| --- | --- | --- | --- | --- | --- |
| IFITM3|13647|AP | -12.0345 | 4.09E-12 | 5.72E-14 | 2.93E-10 | 2.34E-33 |
| PIK3R2|48396|AT | -10.8401 | 0.003596 | 0.0013 | 0.009948 | 2.22E-27 |
| IL1RAP|68106|AT | -10.7 | 1.97E-05 | 2.70E-06 | 0.000143 | 1.02E-26 |
| SOD2|78301|AT | 10.53481 | 154.6042 | 60.52358 | 394.9282 | 5.97E-26 |
| DCN|23655|AT | -10.1902 | 4.75E-09 | 1.19E-10 | 1.89E-07 | 2.19E-24 |
| KDM4C|85826|AT | 9.92235 | 6.44E+18 | 1.24E+15 | 3.35E+22 | 3.33E-23 |
| MYO10|71604|AT | 9.829094 | 6.81E+44 | 7.82E+35 | 5.93E+53 | 8.44E-23 |
| FCER1G|8600|AT | -9.70271 | 1.36E-15 | 1.35E-18 | 1.37E-12 | 2.94E-22 |
| ARHGAP15|55490|AT | -8.88178 | 1.93E-10 | 1.38E-12 | 2.68E-08 | 6.58E-19 |
| NRP1|11195|AT | -8.65214 | 1.08E-18 | 9.18E-23 | 1.27E-14 | 5.05E-18 |
| CARD6|71874|AT | -8.43027 | 3.38E-06 | 1.81E-07 | 6.32E-05 | 3.45E-17 |
| TAGLN|18893|AP | -7.99553 | 0.000183 | 2.21E-05 | 0.001506 | 1.29E-15 |
| TAGLN|18897|AD | -7.71334 | 0.000754 | 0.000121 | 0.004688 | 1.23E-14 |
| FN1|57360|AT | -6.64032 | 7.99E-42 | 5.92E-54 | 1.08E-29 | 3.13E-11 |
| TRAP1|33623|ES | -6.54175 | 4.35E-32 | 1.75E-41 | 1.08E-22 | 6.08E-11 |
| SCPEP1|42600|AA | -6.31493 | 0.000919 | 0.000105 | 0.008048 | 2.70E-10 |
| GLG1|37566|ES | -5.66364 | 7.19E-29 | 1.31E-38 | 3.95E-19 | 1.48E-08 |
| SEPT9|43716|AP | 5.590705 | 4541.187 | 237.1677 | 86952.76 | 2.26E-08 |
| MACF1|1882|ES | -5.04633 | 2.45E-15 | 5.17E-21 | 1.16E-09 | 4.50E-07 |
| ATP1B3|67084|ES | -5.0154 | 0.00793 | 0.001198 | 0.052508 | 5.29E-07 |
| CDC42|1003|AT | -5.01444 | 0.001097 | 7.64E-05 | 0.01574 | 5.32E-07 |
| NDUFA13|48647|AT | -4.93795 | 1.21E-22 | 2.43E-31 | 6.07E-14 | 7.89E-07 |
| TCEA1|83855|ES | -4.59399 | 1.80E-09 | 3.34E-13 | 9.68E-06 | 4.35E-06 |
| NME1-NME2|42504|AP | -4.54436 | 1.64E-19 | 1.29E-27 | 2.07E-11 | 5.51E-06 |
| KRT222|40883|AT | -4.45062 | 0.004346 | 0.000396 | 0.047667 | 8.56E-06 |
| DLGAP4|393774|ME | -4.10794 | 0.027908 | 0.00506 | 0.153922 | 3.99E-05 |
| TPD52L2|60170|ES | -4.015 | 0.061158 | 0.015633 | 0.239254 | 5.94E-05 |
| SSR1|75257|ES | -3.97062 | 0.004567 | 0.000319 | 0.065295 | 7.17E-05 |
| OS9|22692|AT | -3.92362 | 6.72E-29 | 5.68E-43 | 7.95E-15 | 8.72E-05 |
| SH3GLB2|87812|ES | -3.86775 | 0.213483 | 0.097616 | 0.466881 | 0.00011 |
| NRBP1|53009|ES | -3.85915 | 0.000821 | 2.22E-05 | 0.030294 | 0.000114 |
| PACS2|29635|ES | -3.81107 | 0.004781 | 0.000306 | 0.074628 | 0.000138 |
| ARHGAP21|11012|ES | -3.69205 | 0.053407 | 0.011276 | 0.252969 | 0.000222 |
| CHPT1|23996|ES | -3.50367 | 0.000839 | 1.60E-05 | 0.044124 | 0.000459 |
| KDM1A|1030|ES | -3.34412 | 0.009107 | 0.00058 | 0.14301 | 0.000825 |
| CLTA|86331|ES | -2.81753 | 0.050413 | 0.00631 | 0.402807 | 0.004839 |

**Table S2.** AS events involving the signature and the coefficients.

| AS event | Coefficient | P value |
| --- | --- | --- |
| | IFITM3|13647|AP | | --- | | PIK3R2|48396|AT | | IL1RAP|68106|AT | | MYO10|71604|AT | | FCER1G|8600|AT | | ARHGAP15|55490|AT | | CARD6|71874|AT | | SCPEP1|42600|AA | | ATP1B3|67084|ES | | | -14.36485409 | | --- | | -2.800276886 | | -6.699212717 | | 43.3475845 | | 20.3178843 | | -5.249550658 | | -2.389490222 | | 2.111023374 | | 2.953270889 | | | 1.48E-06 | | --- | | 0.000501611 | | 4.46E-07 | | 0.004942072 | | 8.03E-06 | | 0.044076856 | | 0.15716932 | | 0.08612639 | | 0.00790433 | |

**Table S3.** Normal distribution test results of risk score and immune cell infiltration.

| **Tests of Normality** | | | | | | |
| --- | --- | --- | --- | --- | --- | --- |
|  | Kolmogorov-Smirnov^a^ | | | Shapiro-Wilk | | |
|  | Statistic | df | Sig. | Statistic | df | Sig. |
| Risk score | 0.210 | 653 | 0.000 | 0.738 | 653 | 0.000 |
| B cells naive | 0.320 | 653 | 0.000 | 0.508 | 653 | 0.000 |
| B cells memory | 0.322 | 653 | 0.000 | 0.497 | 653 | 0.000 |
| Plasma cells | 0.385 | 653 | 0.000 | 0.300 | 653 | 0.000 |
| T cells CD8 | 0.212 | 653 | 0.000 | 0.771 | 653 | 0.000 |
| T cells CD4 naive | 0.514 | 653 | 0.000 | 0.128 | 653 | 0.000 |
| T cells CD4 memory resting | 0.078 | 653 | 0.000 | 0.943 | 653 | 0.000 |
| T cells CD4 memory activated | 0.485 | 653 | 0.000 | 0.192 | 653 | 0.000 |
| T cells follicular helper | 0.220 | 653 | 0.000 | 0.733 | 653 | 0.000 |
| T cells regulatory (Tregs) | 0.328 | 653 | 0.000 | 0.515 | 653 | 0.000 |
| T cells gamma delta | 0.517 | 653 | 0.000 | 0.208 | 653 | 0.000 |
| NK cells resting | 0.252 | 653 | 0.000 | 0.721 | 653 | 0.000 |
| NK cells activated | 0.125 | 653 | 0.000 | 0.921 | 653 | 0.000 |
| Monocytes | 0.057 | 653 | 0.000 | 0.974 | 653 | 0.000 |
| Macrophages M0 | 0.323 | 653 | 0.000 | 0.532 | 653 | 0.000 |
| Macrophages M1 | 0.204 | 653 | 0.000 | 0.777 | 653 | 0.000 |
| Macrophages M2 | 0.033 | 653 | 0.092 | 0.995 | 653 | 0.053 |
| Dendritic cells resting | 0.440 | 653 | 0.000 | 0.112 | 653 | 0.000 |
| Dendritic cells activated | 0.424 | 653 | 0.000 | 0.234 | 653 | 0.000 |
| Mast cells resting | 0.254 | 653 | 0.000 | 0.737 | 653 | 0.000 |
| Mast cells activated | 0.265 | 653 | 0.000 | 0.692 | 653 | 0.000 |
| Eosinophils | 0.276 | 653 | 0.000 | 0.654 | 653 | 0.000 |
| Neutrophils | 0.304 | 653 | 0.000 | 0.530 | 653 | 0.000 |
|  | | | | | | |

**Table S4.** Normal distribution test results of risk score and immune checkpoints expression.

| **Tests of Normality** | | | | | | |
| --- | --- | --- | --- | --- | --- | --- |
|  | Kolmogorov-Smirnov^a^ | | | Shapiro-Wilk | | |
|  | Statistic | df | Sig. | Statistic | df | Sig. |
| Risk score | 0.210 | 653 | 0.000 | 0.738 | 653 | 0.000 |
| ANGPTL7 | 0.421 | 653 | 0.000 | 0.131 | 653 | 0.000 |
| BTLA | 0.170 | 653 | 0.000 | 0.718 | 653 | 0.000 |
| BTN1A1 | 0.401 | 653 | 0.000 | 0.183 | 653 | 0.000 |
| BTN3A1 | 0.137 | 653 | 0.000 | 0.676 | 653 | 0.000 |
| BTN3A3 | 0.152 | 653 | 0.000 | 0.638 | 653 | 0.000 |
| CD160 | 0.124 | 653 | 0.000 | 0.754 | 653 | 0.000 |
| CD226 | 0.198 | 653 | 0.000 | 0.614 | 653 | 0.000 |
| CD244 | 0.194 | 653 | 0.000 | 0.713 | 653 | 0.000 |
| CD27 | 0.176 | 653 | 0.000 | 0.623 | 653 | 0.000 |
| CD274 | 0.268 | 653 | 0.000 | 0.487 | 653 | 0.000 |
| CD276 | 0.200 | 653 | 0.000 | 0.777 | 653 | 0.000 |
| CD28 | 0.308 | 653 | 0.000 | 0.401 | 653 | 0.000 |
| CD40 | 0.177 | 653 | 0.000 | 0.698 | 653 | 0.000 |
| CD47 | 0.068 | 653 | 0.000 | 0.968 | 653 | 0.000 |
| CD48 | 0.263 | 653 | 0.000 | 0.620 | 653 | 0.000 |
| CD70 | 0.421 | 653 | 0.000 | 0.175 | 653 | 0.000 |
| CD80 | 0.283 | 653 | 0.000 | 0.499 | 653 | 0.000 |
| CD84 | 0.140 | 653 | 0.000 | 0.810 | 653 | 0.000 |
| CD86 | 0.134 | 653 | 0.000 | 0.828 | 653 | 0.000 |
| CD96 | 0.253 | 653 | 0.000 | 0.604 | 653 | 0.000 |
| CTLA4 | 0.452 | 653 | 0.000 | 0.045 | 653 | 0.000 |
| EDA | 0.111 | 653 | 0.000 | 0.804 | 653 | 0.000 |
| EDA2R | 0.186 | 653 | 0.000 | 0.810 | 653 | 0.000 |
| EDAR | 0.301 | 653 | 0.000 | 0.459 | 653 | 0.000 |
| FAS | 0.202 | 653 | 0.000 | 0.665 | 653 | 0.000 |
| FASLG | 0.339 | 653 | 0.000 | 0.343 | 653 | 0.000 |
| HHLA2 | 0.122 | 653 | 0.000 | 0.841 | 653 | 0.000 |
| ICOS | 0.320 | 653 | 0.000 | 0.469 | 653 | 0.000 |
| ICOSLG | 0.214 | 653 | 0.000 | 0.697 | 653 | 0.000 |
| LAG3 | 0.352 | 653 | 0.000 | 0.198 | 653 | 0.000 |
| LAIR1 | 0.168 | 653 | 0.000 | 0.755 | 653 | 0.000 |
| LAIR2 | 0.417 | 653 | 0.000 | 0.117 | 653 | 0.000 |
| LGALS9 | 0.128 | 653 | 0.000 | 0.835 | 653 | 0.000 |
| LILRA2 | 0.138 | 653 | 0.000 | 0.819 | 653 | 0.000 |
| LILRA5 | 0.362 | 653 | 0.000 | 0.302 | 653 | 0.000 |
| LILRA6 | 0.255 | 653 | 0.000 | 0.543 | 653 | 0.000 |
| LILRB1 | 0.168 | 653 | 0.000 | 0.743 | 653 | 0.000 |
| LILRB2 | 0.250 | 653 | 0.000 | 0.542 | 653 | 0.000 |
| LILRB3 | 0.238 | 653 | 0.000 | 0.578 | 653 | 0.000 |
| LILRB4 | 0.125 | 653 | 0.000 | 0.868 | 653 | 0.000 |
| LILRB5 | 0.331 | 653 | 0.000 | 0.363 | 653 | 0.000 |
| LTA | 0.193 | 653 | 0.000 | 0.593 | 653 | 0.000 |
| LTB | 0.194 | 653 | 0.000 | 0.630 | 653 | 0.000 |
| LTBR | 0.188 | 653 | 0.000 | 0.728 | 653 | 0.000 |
| LY9 | 0.248 | 653 | 0.000 | 0.608 | 653 | 0.000 |
| NCR3 | 0.164 | 653 | 0.000 | 0.673 | 653 | 0.000 |
| NCR3LG1 | 0.199 | 653 | 0.000 | 0.736 | 653 | 0.000 |
| NECTIN1 | 0.062 | 653 | 0.000 | 0.958 | 653 | 0.000 |
| NECTIN2 | 0.169 | 653 | 0.000 | 0.855 | 653 | 0.000 |
| NECTIN3 | 0.102 | 653 | 0.000 | 0.813 | 653 | 0.000 |
| NGFR | 0.338 | 653 | 0.000 | 0.330 | 653 | 0.000 |
| PDCD1 | 0.259 | 653 | 0.000 | 0.579 | 653 | 0.000 |
| PDCD1LG2 | 0.242 | 653 | 0.000 | 0.629 | 653 | 0.000 |
| PVR | 0.124 | 653 | 0.000 | 0.887 | 653 | 0.000 |
| PVRIG | 0.151 | 653 | 0.000 | 0.833 | 653 | 0.000 |
| RELT | 0.099 | 653 | 0.000 | 0.744 | 653 | 0.000 |
| SIRPA | 0.082 | 653 | 0.000 | 0.945 | 653 | 0.000 |
| SIRPB1 | 0.238 | 653 | 0.000 | 0.641 | 653 | 0.000 |
| SIRPG | 0.275 | 653 | 0.000 | 0.559 | 653 | 0.000 |
| SLAMF1 | 0.266 | 653 | 0.000 | 0.617 | 653 | 0.000 |
| SLAMF6 | 0.262 | 653 | 0.000 | 0.576 | 653 | 0.000 |
| SLAMF7 | 0.297 | 653 | 0.000 | 0.473 | 653 | 0.000 |
| TIGIT | 0.295 | 653 | 0.000 | 0.412 | 653 | 0.000 |
| TMIGD2 | 0.170 | 653 | 0.000 | 0.783 | 653 | 0.000 |
| TNF | 0.335 | 653 | 0.000 | 0.361 | 653 | 0.000 |
| TNFRSF10A | 0.150 | 653 | 0.000 | 0.745 | 653 | 0.000 |
| TNFRSF10B | 0.107 | 653 | 0.000 | 0.869 | 653 | 0.000 |
| TNFRSF10C | 0.215 | 653 | 0.000 | 0.619 | 653 | 0.000 |
| TNFRSF10D | 0.249 | 653 | 0.000 | 0.484 | 653 | 0.000 |
| TNFRSF11A | 0.143 | 653 | 0.000 | 0.764 | 653 | 0.000 |
| TNFRSF11B | 0.282 | 653 | 0.000 | 0.587 | 653 | 0.000 |
| TNFRSF12A | 0.262 | 653 | 0.000 | 0.656 | 653 | 0.000 |
| TNFRSF13B | 0.289 | 653 | 0.000 | 0.545 | 653 | 0.000 |
| TNFRSF13C | 0.130 | 653 | 0.000 | 0.822 | 653 | 0.000 |
| TNFRSF17 | 0.298 | 653 | 0.000 | 0.372 | 653 | 0.000 |
| TNFRSF14 | 0.174 | 653 | 0.000 | 0.807 | 653 | 0.000 |
| TNFRSF18 | 0.269 | 653 | 0.000 | 0.468 | 653 | 0.000 |
| TNFRSF19 | 0.157 | 653 | 0.000 | 0.789 | 653 | 0.000 |
| TNFRSF1A | 0.154 | 653 | 0.000 | 0.843 | 653 | 0.000 |
| TNFRSF1B | 0.148 | 653 | 0.000 | 0.788 | 653 | 0.000 |
| TNFRSF21 | 0.093 | 653 | 0.000 | 0.912 | 653 | 0.000 |
| TNFRSF25 | 0.133 | 653 | 0.000 | 0.848 | 653 | 0.000 |
| TNFRSF4 | 0.246 | 653 | 0.000 | 0.521 | 653 | 0.000 |
| TNFRSF8 | 0.346 | 653 | 0.000 | 0.357 | 653 | 0.000 |
| TNFRSF9 | 0.300 | 653 | 0.000 | 0.407 | 653 | 0.000 |
| TNFSF10 | 0.186 | 653 | 0.000 | 0.681 | 653 | 0.000 |
| TNFSF11 | 0.406 | 653 | 0.000 | 0.193 | 653 | 0.000 |
| TNFSF12 | 0.077 | 653 | 0.000 | 0.964 | 653 | 0.000 |
| TNFSF13 | 0.148 | 653 | 0.000 | 0.804 | 653 | 0.000 |
| TNFSF13B | 0.265 | 653 | 0.000 | 0.488 | 653 | 0.000 |
| TNFSF14 | 0.351 | 653 | 0.000 | 0.371 | 653 | 0.000 |
| TNFSF15 | 0.221 | 653 | 0.000 | 0.545 | 653 | 0.000 |
| TNFSF18 | 0.337 | 653 | 0.000 | 0.381 | 653 | 0.000 |
| TNFSF4 | 0.312 | 653 | 0.000 | 0.259 | 653 | 0.000 |
| TNFSF8 | 0.162 | 653 | 0.000 | 0.755 | 653 | 0.000 |
| TNFSF9 | 0.193 | 653 | 0.000 | 0.599 | 653 | 0.000 |
| VSIG8 | 0.259 | 653 | 0.000 | 0.485 | 653 | 0.000 |
| VTCN1 | 0.205 | 653 | 0.000 | 0.638 | 653 | 0.000 |
|  | | | | | | |

**Table S5.** Correlation between risk scores and expression of immune checkpoints.

| **Symbol** | **Correlation** | **P-value** | **Symbol** | **Correlation** | **P-value** | **Symbol** | **Correlation** | **P-value** |
| --- | --- | --- | --- | --- | --- | --- | --- | --- |
| **ANGPTL7** | 0.071 | ns | LILRA2 | **0.514** | ******* | **TNFRSF10B** | **0.486** | ******* |
| **BTLA** | 0.175 | *** | LILRA5 | **0.584** | ******* | **TNFRSF10C** | **0.496** | ******* |
| **BTN1A1** | 0.109 | ** | LILRA6 | **0.597** | ******* | **TNFRSF10D** | **0.446** | ******* |
| **BTN3A1** | **0.333** | ******* | LILRB1 | **0.487** | ******* | **TNFRSF11A** | **0.409** | ******* |
| **BTN3A3** | 0.231 | *** | LILRB2 | **0.627** | ******* | **TNFRSF11B** | **0.532** | ******* |
| **CD160** | 0.067 | ns | LILRB3 | **0.635** | ******* | **TNFRSF12A** | **0.655** | ******* |
| **CD226** | **0.431** | ******* | LILRB4 | **0.395** | ******* | TNFRSF13B | 0.020 | ns |
| **CD244** | **0.389** | ******* | LILRB5 | 0.243 | *** | TNFRSF13C | -0.292 | *** |
| **CD27** | **0.316** | ******* | LTA | 0.023 | ns | TNFRSF14 | 0.677 | *** |
| **CD274** | **0.447** | ******* | LTB | 0.296 | *** | TNFRSF17 | 0.157 | *** |
| **CD276** | **0.666** | ******* | LTBR | **0.611** | ******* | **TNFRSF18** | **0.393** | ******* |
| **CD28** | **0.460** | ******* | LY9 | **0.408** | ******* | **TNFRSF19** | **0.454** | ******* |
| **CD40** | **0.642** | ******* | NCR3 | 0.057 | ns | **TNFRSF1A** | **0.704** | ******* |
| **CD47** | 0.130 | *** | NCR3LG1 | **-0.358** | ******* | **TNFRSF1B** | **0.456** | ******* |
| **CD48** | **0.621** | ******* | NECTIN1 | **-0.500** | ******* | TNFRSF21 | -0.229 | *** |
| **CD70** | **0.367** | ******* | NECTIN2 | **0.656** | ******* | TNFRSF25 | 0.112 | ** |
| **CD80** | **0.481** | ******* | NECTIN3 | -0.142 | *** | **TNFRSF4** | **0.398** | ******* |
| **CD84** | **0.310** | ******* | NGFR | 0.189 | *** | TNFRSF8 | -0.001 | ns |
| **CD86** | **0.547** | ******* | PDCD1 | **0.367** | ******* | **TNFRSF9** | **0.470** | ******* |
| **CD96** | **0.492** | ******* | PDCD1LG2 | **0.619** | ******* | **TNFSF10** | **0.482** | ******* |
| **CTLA4** | 0.097 | ** | PVR | **0.446** | ******* | **TNFSF11** | **0.408** | ******* |
| **EDA** | -0.189 | *** | PVRIG | **-0.315** | ******* | **TNFSF12** | **0.433** | ******* |
| **EDA2R** | 0.141 | *** | RELT | 0.157 | *** | **TNFSF13** | **0.437** | ******* |
| **EDAR** | -0.105 | ** | SIRPA | -0.240 | *** | TNFSF13B | 0.238 | *** |
| **FAS** | **0.635** | ******* | SIRPB1 | **0.493** | ******* | **TNFSF14** | **0.410** | ******* |
| **FASLG** | 0.343 | *** | SIRPG | **0.488** | ******* | **TNFSF15** | **0.441** | ******* |
| **HHLA2** | -0.273 | *** | SLAMF1 | **0.518** | ******* | TNFSF18 | -0.115 | ** |
| **ICOS** | 0.436 | *** | SLAMF6 | **0.510** | ******* | TNFSF4 | 0.271 | *** |
| **ICOSLG** | 0.248 | *** | SLAMF7 | **0.382** | ******* | **TNFSF8** | **0.593** | ******* |
| **LAG3** | 0.120 | ** | TIGIT | 0.015 | ns | TNFSF9 | 0.097 | ** |
| **LAIR1** | **0.613** | ******* | TMIGD2 | -0.026 | ns | VSIG8 | 0.044 | ns |
| **LAIR2** | 0.070 | ns | TNF | -0.076 | * | VTCN1 | -0.174 | *** |
| **LGALS9** | **0.462** | ******* | TNFRSF10A | **0.440** | ******* |  |  |  |

Correlation coefficients over 0.3 were in bold. Abbreviation: ns, not significant, “***” means *p*-value < 0.001, “**” means *p*-value < 0.01, “*” means *p*-value < 0.05.
